# Isolated posterior capsule rupture secondary to blunt ocular trauma: Case presentation and literature review

**DOI:** 10.1016/j.ijscr.2024.110116

**Published:** 2024-08-03

**Authors:** Kisato Ichikawa, Akane Tomita, Yumi Suzuki, Shingo Hosoda, Takashi Koto, Makoto Inoue

**Affiliations:** a Kyorin Eye Center, Kyorin University School of Medicine, Tokyo, Japan; b Department of Ophthalmology, National Hospital Organization Saitama Hospital, Japan

**Keywords:** Cataract, Blunt trauma, Posterior capsule rupture

## Abstract

**Introduction:**

An isolated posterior capsule rupture (PCR) is a rare complication associated with traumatic cataracts. We report our findings in three cases of traumatic cataracts with isolated PCR caused by blunt ocular trauma.

**Presentation of cases:**

Case 1: A 1.5-year-old boy was examined after his parents noticed that the center of the pupil of the left eye was white. The mother reported that the boy had fallen and bruised his left forehead 4 months earlier. Case 2: An 18-year-old boy had a traumatic cataract that developed one month after a blow to his eye. Case 3: A 13-year-old boy was treated for hyphema and high intraocular pressure after blunt trauma to his eye. Ten days later, a total cataract developed. Anterior segment optical coherence tomography revealed an isolated PCR with a protruding lens cortex, and ultrasonography showed vitreous opacities.

**Discussion:**

An isolated PCR was observed intraoperatively in the center of the posterior lens capsule. The lens cortex was prolapsed into the PCR or into the vitreous cavity in Case 3. An intraocular lens (IOL) was implanted in the lens capsule or to the ciliary sulcus after vitrectomy in Case 3. Vision improved in all eyes.

**Conclusions:**

Our findings indicated that the external force by a trauma to the eye can lead to an isolated PCR with a protruded lens cortex in young patients. These PCRs can be successfully treated with the IOL implanted in or out of the capsular bag.

## Highlights

• We reported 3 cases of an isolated posterior capsule rupture and cataracts.
• Our cases included a very young patient of 1.5 years of age.
• All three cases were treated with the implantation of an intraocular lens.
• The isolated PCRs developed mainly in teenagers and men in the literature.
• Some cases required surgery immediately in the literature.

## 1 Introduction

An isolated posterior capsular rupture (PCR) associated with a cataract is a rare complication of blunt ocular trauma occurring especially in individuals under the age of 30 [1,2]. It results not only from direct blows to the eye, but also in association with blows to the head during sports activities and playing accidents [[1], [2], [3], [4], [5], [6], [7], [8], [9], [10], [11], [12], [13], [14], [15], [16], [17], [18], [19], [20]].

We report three cases of isolated PCRs associated with trauma and present our findings from the preoperative and intraoperative observations, the surgical procedures and outcomes, and the postoperative course.

## 2 Case presentation

The study has been reported in line with the PROCESS criteria [21].

### 2.1 Case 1

A 1.5-year-old boy was noted by his mother to have a whitening of the center of the pupil of the left eye. The mother reported that the infant had fallen and bruised his left forehead 4 months prior to our examination. Our initial examination found that his best-corrected visual acuity measured with a picture acuity test was at least 20/100 in both eyes. He was very photophobic when the examination light was directed to the left eye.

The eyes were orthophoric with normal corneal reflex, and the intraocular pressure (IOP) was 14 mmHg in both eyes. A hand-held slit-lamp microscope examination showed a total cataract in the center of the lens, and ultrasound tomography showed a convex hyperintense shadow posterior to the lens. The possibility of a rapidly developing cataract was considered, and surgical treatment was performed the day after the initial visit.

**Fig. 1:**
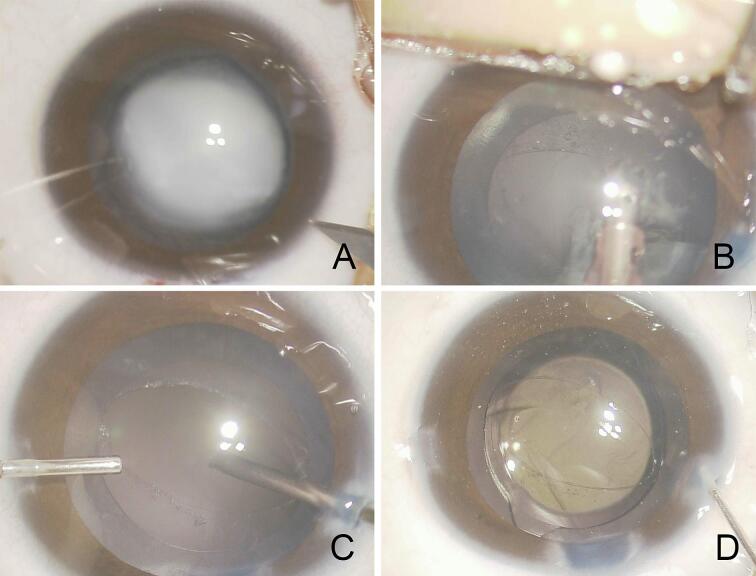
Case 1. Intraoperative images of a 1.5-year-old boy who fell and sustained injury to his forehead. A: Intraoperative image at the beginning of the surgery. The lens opacity is oval with dense central cloudiness. B: During the aspiration of the lens, an oval-shaped posterior capsule rupture is detected. The edge of the ruptured posterior capsule appears to be fibrotic. C: Anterior vitrectomy is performed with a 27-gauge vitreous cutter in a bimanual manner. D: An intraocular lens is implanted in the lens capsule.

The patient underwent surgery under general anesthesia. Continuous curvilinear capsulorhexis (CCC) was completed with forceps, and the lens was aspirated after hydrodelineation without hydrodissection. Intraoperatively, an oval PCR was observed at the center of the posterior lens capsule (Fig. 1). A dehiscence of Zinn's zonules was not observed, and the PCR was localized. An intraocular lens (IOL) with a prospective refraction of +2.0 D was implanted into the lens capsule after anterior vitrectomy. The corneal wounds were closed with 10–0 Nylon sutures. Postoperatively, the patient was treated for potential amblyopia by wearing near-vision glasses with a patch over the unaffected eye for 2 h/day. Ten months after the surgery, a picture acuity test found that the BCVA was 20/63 equally in both eyes.

### 2.2 Case 2

An 18-year-old boy was hit on the right eye by a fist during karate practice resulting in blurred vision. Because the blurred vision did not improve, he visited a local doctor one week later and was diagnosed with a traumatic cataract of the right eye. He was then referred to our clinic.

**Fig. 2:**
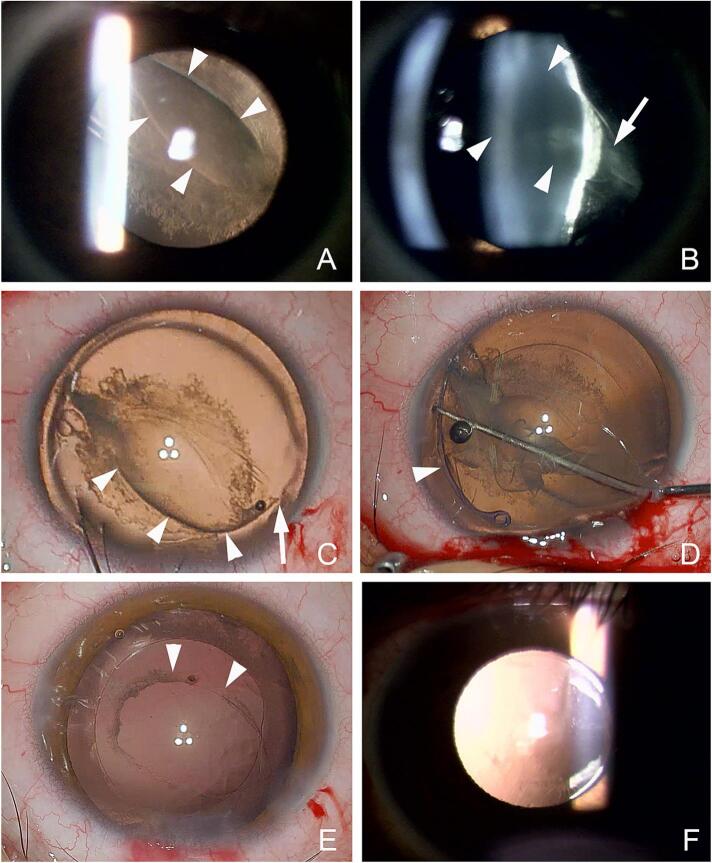
Case 2. Preoperative, intraoperative, and postoperative images of an 18-year-old boy who sustained blunt trauma to his right eye. A: Slit-lamp photographs indicating a longitudinal rupture of the posterior lens capsule with fibrosis around the edge. B: The lens cortex appears to protrude posteriorly from the longitudinal rupture. C: Intraoperative image from the surgeon's view showing that the posterior capsule rupture is consistent with an arcuate fissure (arrowheads) and an outwardly retracted fissure (arrow) without reaching the lens equator and no Zinn's zonule dehiscence. D: After an anterior lens capsulotomy with continuous circular capsulorhexis is completed, a capsular tension ring (arrowhead) is inserted after viscodissection. E: The lens cortex is aspirated and anterior vitrectomy is performed with a 27-gauge vitreous cutter inserted through the ruptured posterior capsule. The fibrous opacity around the edge of the ruptured posterior capsule is also partially removed by the vitreous cutter. The edges of the ruptured posterior capsule do not extend to the lens equator. A one-piece acrylic intraocular lens is implanted into the lens capsule. F: Postoperative slit-lamp photograph at 1 month showing that the intraocular lens is implanted and well-centered.

His BCVA was 20/200 and the IOP was 16 mmHg in the right eye. Slit-lamp biomicroscopy of the right eye at the initial examination revealed an arch-shaped tear of PCR that ran diagonally from 11 to 4 o'clock. The flipped flap of the posterior lens capsule was fibrotic (Fig. 2). The lens cortex protruded posteriorly from the tear of the posterior lens capsule as in eyes with a posterior lenticonus. The reduced visual acuity was diagnosed to be due to the traumatic cataract, and surgery was scheduled one month after the trauma.

Intraoperatively, the PCR was noted not to extend to the lens equator and Zinn's zonule was not dehisced. An anterior lens capsulotomy was completed, and a capsular tension ring (CTR) was inserted without hydrodissection to maintain lens capsular support. The lens was aspirated and anterior vitrectomy was performed with a 27-gauge vitreous cutter through the site of the PCR in a bimanual manner. The fibrous opacity around the PCR was also partially removed by the vitreous cutter, and a one-piece acrylic IOL was implanted into the lens capsule. After 2 months, the BCVA improved to 20/18 with a well-centered IOL.

### 2.3 Case 3

The left eye of a 13-year-old boy was injured with a wooden stick while playing with a friend and was examined at a local clinic. He had no special medical history. Vision was light perception due to a hyphema in the left eye, and the patient was treated conservatively with topical dexamethasone eye drops and oral acetazolamide. One week later, the hyphema had disappeared, and a total cataract was observed.

**Fig. 3:**
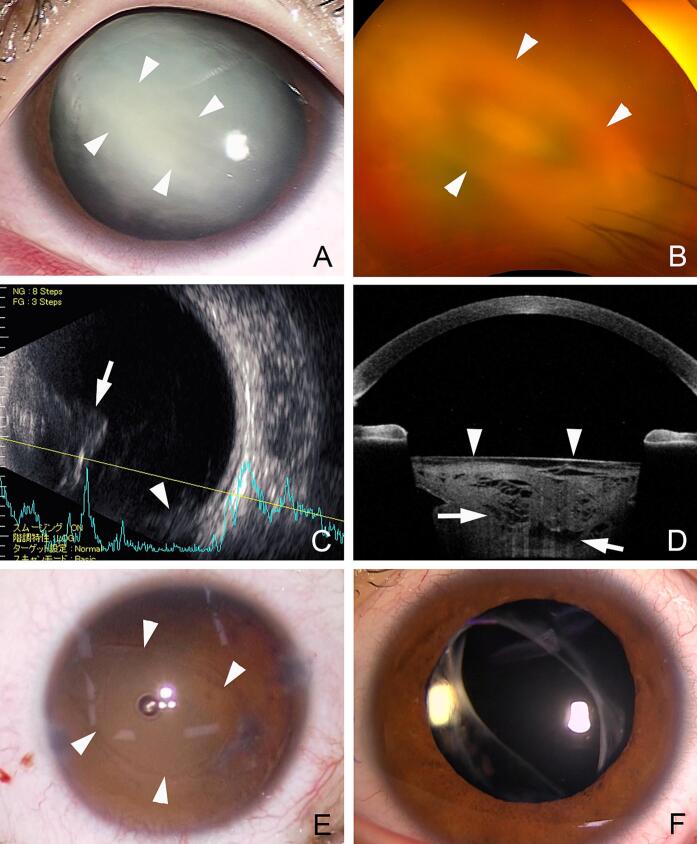
Case 3. Preoperative, intraoperative, and postoperative images of a 13-year-old boy. A: Slit-lamp photographs showing a central lens opacity with an oval-shaped dense opacity (arrowheads). B: Ultra-widefield fundus photograph showing an oval-shaped lens opacity. The lens cortex appears to protrude posteriorly through the longitudinal rupture. C: Ultrasound image showing a protruded opacity (arrow) behind the lens and an opacity at the bottom of the vitreous cavity (arrowhead). D: Anterior segment optical coherence tomographic image showing that the anterior surface of the lens capsule is flattened (arrowheads) and lens opacity is not uniform with partial extrusion (arrows). E: After the lens is removed, the ruptured posterior capsule is oval-shaped (arrowheads). The superior-nasal edge of ruptured posterior capsule appears to reach to the equator. F: Postoperative image indicates that the IOL is implanted in the center with optic-captured position.

At our initial examination, the visual acuity in the left eye was hand motion and the IOP was 26 mmHg. Slit-lamp examination showed a total cataract with opacities around a tear of the posterior lens capsule (Fig. 3). Ultrasound B-mode images showed opacities protruding posterior to the lens and vitreous opacities which we suspected as being lens materials that had fallen into the vitreous cavity. Anterior segment optical coherence tomography (CASIA2, TOMEY Corp, Nagoya) showed a flattening of the anterior lens surface and the lens body was not uniform. Based on these findings, it was assumed that the lens contents had prolapsed into the vitreous cavity through the PCR, and surgery was scheduled.

After removal of the lens cortex, a PCR was detected at the center of the posterior lens capsule, and the tear of the PCR at 4 o'clock extended to the lens equator. Because observation of the fundus was difficult due to vitreous opacities, a three-port 25-gauge vitrectomy was performed to remove the opacities. There was no retinal detachment and no obvious retinal abnormalities. Because the PCR extended to the lens equator, a 3-piece acrylic IOL was inserted into the ciliary sulcus above the lens capsule, and the optics of the IOL was placed in the capsular bag. Postoperatively, the vision recovered to 20/18.

## 3 Discussion

We have presented our findings in 3 cases of cataracts associated with an isolated PCR that developed after a blunt trauma in a 1.5-, 18-, and 16-year-old boys. In all 3 cases, there was no dehiscence of the Zinn's zonule, and the oblique tear of the posterior capsule extended from the center to the rim of the posterior lens capsule. The lens material protruded through the PCR into the vitreous cavity and caused visual disturbances.

The findings in 32 eyes in the previous reports including our 3 eyes that developed a PCR from a traumatic event are presented in Table 1 [[1], [2], [3], [4], [5], [6], [7], [8], [9], [10], [11], [12], [13], [14], [15], [16], [17], [18], [19], [20]]. The ages ranged from 1.5 to 71 years, and 4 patients were <10-years-of-age, 16 eyes (50 %) were 10 to 20 years, and 10 eyes were >20 years. Thus, the incidence of PCR was highest in the 10 to 20 years group. The sex distribution was 30 men (94 %) and 2 women indicating a male-dominated distribution. The time to surgery varied from 1 day to 2 years from the time of the trauma. The time to surgery was <1 month in 17 eyes (53 %), 1 to 3 months in 8 eyes, >3 months in 6 eyes, and unknow in 1 eye. These findings indicated that the acute type of PCR develops lens opacity quickly while the chronic type develops slowly. The shape of PCR was oval in 6 eyes (19 %) and either longitudinal, linear, or vertical in 8 eyes (25 %). In eyes that had surgery within one month of the trauma, the preoperative vision was ≤20/200 in 8 eyes (62 %), and >20/200 in 5 eyes. These findings indicated that the acute type had a greater reduction of vision. In eyes that had surgery from one to 3 months, the preoperative vision was ≤20/200 in 4 eyes and >20/200 in 3 eyes. In eyes that had the surgery >3 months after the trauma, the preoperative vision was ≤20/200 in 2 eyes and >20/200 in 4 eyes (67 %). The reduced vision in the acute type was due to lens opacities including lens fragments extruded into the vitreous cavity as in Case 3 [15]. In contrast, the reduced vision in the chronic type was due to increased distortions caused by the extruded lens cortex through the posterior capsule window similar to Case 2 [9,20].

**Table 1:** Characteristics of isolated posterior capsule rupture (PCR) reported in the literature.

Authors (year)	Age(years)/sex	Trauma	Time to surgery	PCR shape	Surgery	IOL fixation	Visual acuity	
							Initial	Final
Saika S, 1997 [3]	19/M	Struck by golf ball	12 days	–	Aspiration	Posterior chamber	–	20/20
Campanella, 1997 [4]	8/M	Blunt trauma	8 weeks	Oval	ECCE and A-vit	Posterior chamber	HM	20/20
Campanella, 1997 [4]	13/M	BB gun pellet	8 weeks	Oval	ECCE and A-vit	Posterior chamber	CF	20/15
Thomas R, 1998 [5]	32/M	Fist	6 weeks	–	ECCE and A-vit	Posterior chamber	HM	20/20
Thomas R, 1998 [5]	10/M	Stick	8 weeks	–	ECCE and A-vit	Posterior chamber	20/120	20/20
YasukawaT, 1998 [6]	11/M	Eraser	20 days	–	PPL	Aphakia	20/100	20/20
Pavlovic S, 2000 [7]	23/M	–	6 days	–	PPL and PPV	Sulcus	HM	20/20
Rosen WJ, 2000 [8]	18/M	Paint pellet	1 week	Curvilinear	PPL and PPV	Sulcus	HM	20/30
Lee SI, 2001 [9]	25/M	Rubber rope	3 days	Longitudinal	PEA and A-vit	Capsular bag	20/200	20/20
Li KK, 2005 [10]	15/M	Brick	2 weeks	Linear tear	Aspiration and A-vit	Capsular bag	CF	20/20
Pushker N, 2005 [11]	11/F	–	2 months	Vertical tear	Aspiration and A-vit	Optic capture	20/125	20/20
Por YM, 2006 [12]	19/M	–	2 months	–	Aspiration	Capsular bag	–	–
Grewal DS, 2009 [13]	11/M	Projectile from a slingshot	2 days	Longitudinal	PEA	Capsular bag	20/400	20/20
Georgalas I, 2011 [14]	71/F	Falling and striking eye	9 months	–	PEA+PPV	Sulcus	20/30	20/40
Mansour AM, 2014 [2]	6/M	Slipped and hit forehead	1 day	Oval	Aspiration and A-vit	In sulcus	HM	20/20
Matalia J, 2015 [15]	9/M	Cricket ball	4 weeks	oval	Aspiration and A-vit	Posterior chamber	20/100	–
Choudhary N, 2016 [16]	15/M	Cricket ball	–	–	Aspiration and A-vit	Posterior chamber	LP	20/20
Wan W, 2017 [1]	18/M	Stick	5 days	Transverse	Aspiration and A-vit	Sulcus	CF	20/20
Wan W, 2017 [1]	33/M	Chair	2 years	Longitudinal	Aspiration	Capsular bag	10/20	20/25
Wan W, 2017 [1]	47/M	Wood	1 year	Longitudinal	Aspiration	Capsular bag	HM	20/25
Wan W, 2017 [1]	33/M	Stone	17 days	Longitudinal	Aspiration	Capsular bag	HM	20/25
Srinivasaraghavan P, 2018 [17]	26/M	log	4 weeks	oval	PEA and A-vit	capsular bag	?	20/20
Jain P, 2019 [18]	15/M	Wooden stick	5 weeks	Concentric	PEA	Capsular bag	CF	20/30
Prager AJ, 2020 [19]	68/M	Baseball	2 weeks	Wedge-shaped tear	PEA (femtosecond laser-assisted) and A-vit	Sulcus	20/200	20/30
Elksnis Ē, 2021 [20]	39/M	Rubber belt	3 weeks	Longitudinal	PEA+PPV	Reverse optic capture	20/40	20/16
Case 1	1.5/M	Slipped and hit forehead	4 months	Oval	Aspiration	Capsular bag	20/100	20/63
Case 2	18/M	Fist	1 month	Longitudinal	Aspiration with CTR and A-vit	Capsular bag	20/200	20/18
Case 3	13/M	Wooden stick	1 week	Oval	Aspiration and PPV	Sulcus	HM	20/19

IOL = intraocular lens, ECCE, HM = hand motion, CF = counting fingers, A-vit = anterior vitrectomy, PPL = pars plana lensectomy, PPV = pars plana vitrectomy, PEA = phacoemulcification and aspiration, LP = light perception, CTR = capsular tension ring.

Campanella and associates hypothesized that an isolated PCR was caused by an extension of the posterior capsule in the centrifugal direction caused by a tractional force on the posterior capsule by Wieger's ligament [4]. The external force from the blunt trauma pushes the anterior lens capsule posteriorly in the centrifugal direction. This was due to an increase in the internal pressure of the lens, and the isolated PCR develops in the center of the posterior lens capsule resulting in the prolapse of the lens cortex. This explains why the lens opacity in Case 2 was limited to the area around the isolated PCR. The reduced vision was caused by the irregular shaped lens.

In Case 1 and 2 where the PCR did not extend to the lens equator, an IOL was implanted in the lens capsule. In Case 2, a CTR was inserted before the aspiration and removal of the lens cortex to stabilize the lens capsule. The IOL was implanted successfully with good results [20]. In Case 3 where the isolated PCR extended to the equator, it was better to use sulcus fixation with optic capture of the IOL due to the risk of IOL deviation and subluxation. A subluxation of the lens cortex into the vitreous cavity and retinal detachment due to the trauma was suspected. Thus, pars plana vitrectomy was performed to remove lens cortex that had subluxated into the vitreous cavity. We recommend that special precautions should be taken during the examination and management of such traumatic cases for the possibility of a PCR especially preoperatively.

## 4 Conclusions

An isolated PCR caused by blunt ocular trauma in young individuals was found at the center of the posterior lens capsule, and the lens cortex can prolapse into the isolated PCR. Because of the mechanism for the development of a PCR, damage to the Zinn's zonule was expected to be limited with good support of the lens capsule at the equator, the intraocular lens should be implanted in the lens capsule or in the optic captured position.

## Consent

1. Written informed consent was obtained from the patient for publication and any accompanying images. A copy of the written consent is available for review by the Editor-in-Chief of this journal on request.
2. Written informed consent was obtained from the patient's parents/legal guardian for publication and any accompanying images. A copy of the written consent is available for review by the Editor-in-Chief of this journal on request.

## Disclosure

The authors indicate no government or nongovernment financial support was involved in the work for this submission.

## Ethical approval

Ethical approval by the Ethics Committee of Kyorin University School of Medicine was not necessary as the format of this paper is a case report.

## Funding

No funding was provided for this case report.

## Author contribution

Kisato Ichikawa: data analysis, interpretation, writing the paper.

Akane Tomita: data analysis, interpretation.

Yumi Suzuki: data analysis, interpretation, supervision.

Shingo Hosoda: data collection, interpretation.

Takashi Koto: data analysis, interpretation, supervision.

Makoto Inoue: study concept or design, data collection, data analysis, interpretation, writing the paper, supervision.

## Guarantor

Makoto Inoue, MD.

## Research registration number

This report does not include any ‘First in Man’ studies.

## Conflict of interest statement

The authors have no conflicts of interest to report. No funding has been given for his study.
